# Clinicians’ Social Support, Job Stress, and Intent to Leave Healthcare during COVID-19

**DOI:** 10.3390/healthcare10020229

**Published:** 2022-01-26

**Authors:** Ayhan Tabur, Avishek Choudhury, Abdurrahim Emhan, Cengiz Mengenci, Onur Asan

**Affiliations:** 1 Emergency Department, Gazi Yaşargil Training and Research Hospital, Diyarbakir 21070, Turkey; ayhan.tabur@saglik.gov.tr; 2 Stevens Institute of Technology, School of Systems and Enterprises, Hoboken, NJ 07030, USA; achoudh7@stevens.edu; 3 Collage of Business Administration, University of Central Florida, Orlando, FL 32816, USA; aemhan@gmail.com; 4 Department of Quality, Bower Hospital, Diyarbakir 21100, Turkey; cengizmengenci@yahoo.com

**Keywords:** COVID-19, occupational stress, social support, workload, burnout

## Abstract

The onset of COVID-19 has escalated healthcare workers’ psychological distress. Multiple factors, including prolonged exposure to COVID-19 patients, irregular working hours, and workload, have substantially contributed to stress and burnout among healthcare workers. To explore the impact of COVID-19 on healthcare workers, our study compares the job stress, social support, and intention to leave the job among healthcare workers working in a pandemic (H_P_) and a non-pandemic hospital (H_NP_) in Turkey during the pandemic. The cross-sectional, paper-based survey involved 403 healthcare workers including physicians, registered nurses, health technicians, and auxiliary staff across two hospitals from 1 September 2020 to 31 November 2020. The findings indicate a significant impact of ‘Job stress’ on ‘Intent to leave’ job among participants in the H_P_. We noted that ‘intent to leave’ and ‘job stress’ were significantly higher among the H_P_ healthcare workers than those working in the H_NP,_ respectively. However, workers’ ‘social support’ was significantly lower in the H_P_. Healthcare workers, during COVID-19, face several hurdles such as job stress, reduced social support, and excessive workload, all of which are potential factors influencing a care provider’s intent to leave the job.

## 1. Introduction

As the COVID-19 pandemic continues, several studies have discussed the major paradigm shifts in the utilization of healthcare services [1,2] and hindered care, particularly in places hit hard by the pandemic [3]. Due to the unforeseen impact of COVID-19 on the global healthcare system and insufficient resources, many people have missed out on essential care, including interventions for cancer [4]. Although the deterring effect of COVID-19 was noted globally, healthcare establishments of developing nations, particularly, suffered the most [5].

It was on 11 March 2020 when Turkey’s Ministry of Health announced their first known coronavirus case. Since then, there has been a surge in COVID-19 deaths [6]. As of June 2021, approximately 5,336,073 confirmed cases of COVID-19 with 48,795 deaths had been reported to the World Health Organization [6]. The sudden surge in patients has not only led to a shortage in personal protection equipment (PPE) but also imposed an excessive workload on the national healthcare system. As most hospitals were on the verge of maximum capacity, in several healthcare establishments in Turkey, clinicians had to choose between patients. The nation also experienced a shortage of medications and staff, such that COVID-19 patients were given medication three to four days late [7]. The surge in COVID-19 also resulted in the death of healthcare workers. Doctors held a protest from 13 to 20 September 2020 to commemorate Turkish health workers who died from COVID-19 [8].

The onset of COVID-19 significantly escalated clinicians’ psychological distress [9,10]. When COVID-19 reached Turkey, news reports of its rapid spread and fatalities further intensified the level of stress of clinicians—impeding their physical and mental health [11]. During the pandemic, multiple factors such as working in higher-risk units, inadequate training, inadequate compliance with safety precautions, prolonged exposure to COVID-19 patients, irregular working hours, and increased workload have substantially contributed to stress and burnout among healthcare workers [12,13,14]. COVID-19 has also forced healthcare workers to stay away from their families and friends. A recent study also demonstrated that healthcare workers working at the COVID-19 units have an increased need for psychological support [15]. Prolonged workload and burnout may lead healthcare workers to quit their jobs, particularly in low- and middle-income nations.

Developing nations with limited resources suffered the most from COVID-19. Such countries experienced a shortage of human resources as care providers were floated from clinical practice to join COVID-19 task forces [16]. Scarcity of PPE, an increased workload, inadequate training, infection control practices, and pandemic fatigue added to the existing problems of care providers. In most of the countries, including developed nations, all hospitals cared for COVID-19 patients, so their entire staff were under continuous pressure and experienced continuous burdensome and burnout. However, in some regions of Turkey, the local authorities designated some hospitals as either pandemic hospitals or non-pandemic hospitals. It would be interesting to see how “job stress” and “intention to leave the job” differ among healthcare workers between those two hospitals to better understand the impact of COVID-19 on healthcare workers.

During this type of crisis, we must comprehend the challenges faced by healthcare workers. Increasing job stress and deterring social support may induce burnout and other forms of work-related psychological distress. Therefore, understanding the emotional needs of healthcare workers is essential [17]. This study investigated and compared job stress, social support, and intention to leave the job among healthcare workers attending COVID-19 patients versus the ones serving non-COVID-19 patients in two different hospitals in Turkey.

## 2. Materials and Methods

### 2.1. Study Design

This study was approved by the internal review board of Gazi Yaşargil Education and Research Hospital’s Ethics Committee (IRB ID 521). The study team surveyed two hospitals in Turkey to capture the impact of COVID-19 on constructs such as job stress, social support, and intent to leave. One hospital (non-pandemic: H_NP_) was kept secluded from COVID-infected patients, and the other hospital (pandemic: H_P_) was exclusively involved in treating COVID-infected patients. The hospital’s seclusion was initiated in March 2020, soon after Turkey’s Ministry identified the first coronavirus case. Our data collection started on 1 September and continued through 31 November 2020, during which a paper-based survey was randomly distributed to all healthcare workers in both the hospitals. The participants gave verbal consent before filling out the surveys.

This study used a paper-based, cross-sectional survey, which was distributed by the research team to the participants and collected by the research team from participants’ offices. The survey responses were then compared to measure the significant difference between healthcare workers in pandemic and non-pandemic hospitals. Note that participants were not involved in our research’s design, conduct, reporting, or dissemination plans.

### 2.2. Instrumentation

The study employed a survey questionnaire adopted from various scales and consisted of 55 items. All survey questions were modified and adapted to fit the context of our research. The survey was designed to assess healthcare workers’ *social support*, *job stress*, and *intent to leave* the job. It also recorded respondents’ demographic information such as gender, age, marital status, education status, marital status, job position, work experience, working hours that extending into overtime (overtime hrs/week), and department of work.

The study survey included all Social Support Scale (SSS) questions to measure social support. The SSS, a 7-point Likert scale developed by Zimet et al. (1988), aims to determine the social support elements as perceived by individuals [18]. The Cronbach’s alpha coefficient of 0.92 indicated the internal reliability of the SSS. The questions capture three different aspects of social support: (a) family, (b) friends, and (c) special person. The score obtained from any subscales (friend, family, or special person) may range between 4 and 28, and the total score (summation of subscales) may vary between 12 and 84, where a higher score indicates higher perceived social support.

To assess the level of job stress, the study used all questions from a well-established Job Stress Scale (JSS) with internal reliability of 0.83. The JSS was developed by Cohen and Williamson (1988) [19]. It is a 5-point Likert scale that consists of fourteen items.

Lastly, to determine the intention to leave the job, our study leveraged the results of the validated Turnover Intention Scale (TIS) with an internal reliability of 0.81. The TIS, developed by Walsh et al. (1985), is a 5-point Likert scale with eight items [20].

### 2.3. Statistical Data Analysis

We calculated descriptive statistics on sociodemographic and study variables. Participants’ responses were added to develop a total score. We used Levene’s test to ensure the two groups’ homogeneity of variance (*p* > 0.05). Since the measures including healthcare workers’ job stress, social support, and intention to leave the job can depend on several external factors, we controlled all our analyses for age, working hours (overtime h/week), sex, education level, marital status of participants, work experience, and place of work (clinical department). We then calculated the adjusted means of the dependent study variables for the two hospital groups and compared them across two hospitals (H_NP_ and H_P_) using the one-way analysis of covariance (ANCOVA). We also used hierarchical regression to capture the effect of ‘Job stress’ and ‘Social support’ on participants’ ‘Intent to leave’ job, separately, in both the hospital groups. All statistical analyses were conducted using SPSS Version 27 (IBM Corp, Armonk, NY, USA )

## 3. Results

The survey was distributed to 700 healthcare workers, of which 418 people responded. After discarding incomplete responses, 403 complete surveys were obtained, as shown in Table 1. Note that not all participants (*n* = 403) completed the demographic questions. Most of the participants in the pandemic hospitals were registered nurses (45%), followed by auxiliary staff (26.5%), and clinicians (17.2%). The non-pandemic hospital majority of the participants were auxiliary staff (35.4%), followed by health technicians (30.4%), and registered nurses (17.4%).

According to Levene’s test of equality of error variance, all measures (dependent variables) in the study failed to reject the null hypothesis that the error variance of the dependent variable is equal across groups (intent to leave: *p* = 0.051; job stress: *p* = 0.660; social support: *p* = 0.498). Table 2 shows the comparison of study variables between the H_P_ and H_NP_. We noted that the ‘Intent to leave’, ‘Social support’, and ‘Job stress’ were significantly different between the participants in the H_P_ and H_NP_ (intent to leave: *p* = 0.001; job stress: *p* < 0.001; social support: *p* = 0.006). The analysis also controlled for confounding factors including participant’s ‘Age’, ‘Working hours’, ‘Education level’, ‘Sex’, ‘Work experience’, ‘Marital status’, and ‘Working place.’ The control variables were used as covariates in the analysis. ‘Education level’ and ‘Working place’, had a significant impact on participants’ ‘Intent to leave’ job. Additionally, the analysis also captured the significant impact of ‘Marital status’ on participants’’ ‘Social support’ and ‘Working place’ on their ‘Job stress’ and ‘Social support’. No other significant relationships were observed.

Table 3 shows the original and adjusted means of the dependent study variables. Table 2 and Table 3 together indicate that the adjusted means of ‘Intent to leave’ and ‘Job stress’ were significantly higher among the healthcare providers in the H_P_. However, ‘Social support’ was noted to be significantly lower in the H_P_.

We also performed a hierarchical regression, controlling for all confounding variables, to capture the impact of ‘Job stress’ and ‘Social support’ on ‘Intent to leave’, separately, for the two hospital groups. For the H_P_, ‘Job stress’ was the significant predictor of ‘Intent to leave’ (Beta = 0.325, *p* = 0.003). No significant effect of ‘Social support’ was noted (Beta = −0.122, *p* = 0.055). Similar findings were also observed in the H_NP_. No significant effect of ‘Social support’ was observed (Beta = −0.007, *p* = 0.812). However, ‘Job stress’ was a significant predictor of ‘Intent to leave’ (Beta = 0.196, *p* < 0.001).

Since ‘Education level’ and ‘Working place’ were significant in Table 2, we also captured their impact on ‘Intent to leave’, ‘Job stress’, and ‘Social support’, separately for both the hospital groups. The analyses were controlled for all other variables. In the H_P_ (R-squared = 0.191, *p* < 0.001)_,_ ‘Education level’ was a significant predictor of ‘Intent to leave’ (Beta = −0.204, *p* = 0.003). No other significance was found. In the H_NP_ (R-squared = 0.339, *p* < 0.001) ‘Education level’ and ‘Working place’ were significant predictors of ‘Intent to leave’ (Beta = −0.170, *p* = 0.030; Beta = 0.048, *p* = 0.048, respectively). In the non-pandemic group (R-squared = 385, *p* < 0.001), ‘Working place’ was also a significant predictor of ‘Job stress’ (Beta = 0.190, *p* = 0.013). No other significance was observed.

## 4. Discussion

This is one of the first studies conducted during the peak time of COVID-19 in a developing country which has one of the highest COVID-19 infection and mortality rates in the world in 2020. The findings of our study comparing a pandemic and non-pandemic hospital during the peak time of COVID-19 has several insights. Note that most of the hospitals care for pandemic patients in most countries; however, in this study we had the chance to compare a pandemic and non-pandemic hospital. Our study shows that the ‘intent to leave’ and ‘job stress’ were significantly higher among healthcare workers attending COVID-19 patients compared to healthcare workers in a non-pandemic hospital. More importantly, our study noted that ‘social support’ among healthcare workers in the pandemic hospital was significantly lower than those in the non-pandemic hospital. Additionally, the study also showed that job stress was significant predictor of ‘intent to leave’ in both settings; however, ‘social support’ did not have a significant impact on ‘intent to leave’. Healthcare workers with higher levels of education were also less likely to leave their job in both the hospital groups. However, healthcare workers with more working hours (overtime/week) in the pandemic hospital were less likely to leave the job. Finally, working place within the hospital also had a significant impact on ‘job stress’ and ‘intent to leave’.

In times of crisis such as COVID-19, healthcare workers continue to work, despite the health risks, isolation from families, and hurdles, including an increased influx of critically ill patients, job stress, insufficient resources, and a frequent need for overtime. Such escalating workload is compounding the already demanding work schedules (i.e., 12 h shifts, night shifts) of care providers [21,22]. Currently, healthcare workers in many developing nations are facing challenges taking mandated work breaks and recovery from fatigue. Fatigue at work can jeopardize care providers’ health by making them susceptible to burnout and psychological distress [23,24].

Job stress and excessive workloads have been a long-standing concern of the healthcare industry, even without any crisis or pandemic [25]. However, during the COVID-19 pandemic, these occurrences became prominent, mainly due to the sudden spike in healthcare workers’ workload that resulted from increased patient inflow, the uncertainty of COVID-19 treatment plans, and the isolation policy, which calls for condensed outpatient workload in all hospitals except COVID-19 triage and isolation hospitals [26]. The onset of COVID-19 further escalated the existing stress and cognitive and physical challenges of care providers worldwide. The subsequent and escalating demand that has been placed on the overall healthcare system since 2020 has been negatively impacting care providers’ social support and job stress and consecutively encouraging them to quit their jobs [27]. Consistent with recent literature [26,28], evidence from our study shows that ‘Job stress’ was higher in healthcare workers working in the pandemic hospital than healthcare workers working in non-pandemic hospitals. Care providers such as nurses and doctors, in general, are exposed to excessive workloads and health risks, which are increased among the frontline nurses working in COVID-19 hospitals.

Persistent job stress may also impede job satisfaction and encourage care providers to quit their jobs [29]. As noted in our study, ‘job stress’ significantly predicts intent to leave. Furthermore, healthcare providers in the pandemic hospital had significantly higher ‘Intent to Leave’ jobs and ‘job stress’ than non-pandemic hospital workers. Similar findings were also reported during the 2003 severe acute respiratory syndrome (SARS) outbreak, where care providers exhibited reluctance to work and contemplated resignation [30]. After the outbreak of SARS, care providers also reported chronic stress effects for months to years [31]. Health concerns such as anxiety, depression, burnout, insomnia, and post-traumatic stress disorder are often common challenges faced by care providers and can impact job stress, social support, and intent to leave. Even during regular working conditions and workload, approximately 33% of nurses and 45% of physicians serving critically ill patients suffer from severe burnout syndrome [32,33]. According to a study in China, care providers treating COVID-19 patients reported high rates of depression (50%), anxiety (45%), and distress (72%) [9]. Other studies from Italy and France also reported a high percentage of post-traumatic stress disorder and burnout among nurses treating COVID-19 patients [34,35].

Our study also noted that healthcare workers in the pandemic hospital had significantly less social support. There might be several reasons for this finding. During the peak time of the pandemic in 2020, there was a high infection and mortality rate even among the healthcare workers in Turkey since the vaccines were not available until early 2021. Most of the healthcare workers isolated themselves from their families and friends to protect them. This was a learning process for global healthcare, and some countries tried to do wide range of campaigns to support and moralize frontline healthcare workers since this is critical to improve the mental health of healthcare workers during this crisis time. For instance, several studies have captured the impact of social support on mental health on care during COVID-19 [35,36,37]. A recent study in China reported the positive impact of social support on the mental health of care providers [38]. Another study in Italy noted that care providers with higher social support had a significant reduction in burnout symptoms [35].

Lastly, according to our findings, healthcare workers with higher levels of education were also less likely to leave their job in both the hospital groups. In other words, individuals with lower levels of education had higher intent of leaving their jobs. The finding is in line with the literature that shows higher turnover rates among technicians and nurses compared to physicians and doctors [39,40]. Currently, hospitals worldwide are paying incentives to front-line healthcare workers for doing overtime, and the incentives are acting as extrinsic motivation for some healthcare staff.

### Limitations

Our study provides the current understanding of job stress, social support, and intent to leave among healthcare workers in pandemic and non-pandemic hospitals in Turkey. A few limitations of this study must be acknowledged. Although our findings are limited to two specific hospitals in Turkey, the findings and insights are supportive of healthcare systems worldwide. Moreover, the cross-sectional study measures healthcare workers’ self-reported perception of job stress, social support, and intent to leave via a paper survey at a given time frame, assuming honest and correct responses from all participants.

## 5. Conclusions

Our study shows that the healthcare workers in a pandemic hospital experienced significantly higher job stress and intent to leave compared to healthcare workers in a non-pandemic hospital. The study also showed that healthcare workers in the pandemic hospital received significantly less social support from their families as well as friends. During this pandemic, the healthcare industry is experiencing a significant shortage of frontline workers globally, and managing job stress can increase frontline workers’ intent to work. The healthcare industry should also be more prepared and find alternative ways to support healthcare workers in times of crisis and isolations.

## Figures and Tables

**Table 1:** Participant demographics.

Variables	Pandemic Hospital (H_P_)	Non-Pandemic Hospital (H_NP_)
	*n* (%)	*n* (%)
**Gender**		
Male	138 (57.7)	78 (47.6)
Female	101 (42.3)	85 (51.8)
**Age**		
20–30	89 (37.2)	95 (57.9)
31–40	97 (40.6)	43 (26.2)
41–50	49 (20.5)	22 (13.4)
50+	4 (1.7)	4 (2.4)
**Marital Status**		
Married	151 (64.0)	71 (44.9)
Single	85 (36.0)	87 (54.9)
**Education**		
High School	31 (13.0)	20 (12.3)
University	48 (20.2)	61 (37.4)
Graduate Education	159 (66.5)	82 (50.3)
**Job Position**		
Physician	41 (17.2)	27 (16.8)
Nurse	107 (45.0)	28 (17.4)
Health Technician	27 (11.3)	49 (30.4)
Auxiliary Staff	63 (26.5)	57 (35.4)
**Work Experience**		
1–5 years	71 (30.2)	75 (47.5)
6–10 years	69 (29.4)	32 (20.3)
11–15 years	43 (18.3)	22 (13.9)
16–20 years	31 (13.2)	23 (14.6)
21+ years	21 (8.9)	6 (3.8)
**Working Hours (Overtime)**		
1–5 h/week	55 (23.0)	61 (37.2)
6–10 h/week	47 (19.7)	38 (23.2)
11–15 h/week	40 (16.7)	26 (15.9)
16+ hours/week	97 (40.6)	39 (23.8)
**Working Place**		
Services	98 (41.9)	28 (18.1)
Emergency	80 (34.2)	30 (19.4)
Diagnosis-Examination	11 (4.7)	38 (24.5)
Everywhere	45 (19.2)	59 (38.1)

Note: ‘Work experience’ refers to the number of years participants were employed in the respective hospitals and ‘Working hours’ indicates the number of hours participants worked overtime per week during the course of this study.

**Table 2:** One-way ANCOVA: Comparing pandemic and non-pandemic hospitals.

	Intent to Leave		Job Stress		Social Support	
	F	Sig	F	Sig	F	Sig
**Hospital type (H_P_~H_NP_)**	10.872	0.001 **	45.053	<0.001 ***	7.734	0.006 **
**Age (years)**	0.017	0.896	0.320	0.572	0.002	0.964
**Working hour (overtime hrs/week)**	1.126	0.289	1.544	0.215	2.884	0.090
**Education level**	18.947	<0.001 ***	2.714	0.100	5.116	0.024
**Sex**	3.168	0.076	0.060	0.806	0.520	0.471
**Work experience**	0.265	0.607	1.848	0.175	0.255	0.614
**Marital status**	0.596	0.441	0.001	0.981	4.556	0.033 *
**Working place**	10.872	0.001 **	45.053	<0.001 ***	7.734	0.006 **

* Significant at alpha < 0.05; ** Significant at alpha < 0.01; *** Significant at alpha < 0.001. Note: ‘Work experience’ refers to the number of years participants were employed in the respective hospitals and ‘Working hours’ indicate the number of hours participants worked overtime per week during the course of this study.

**Table 3:** Adjusted and original means of dependent variables across the pandemic and the non-pandemic hospital.

	Intent to Leave		Job Stress		Social Support	
	Mean	Adjusted Mean	Mean	Adjusted Mean	Mean	Adjusted Mean
**Non-pandemic hospital**	8.344	8.329	34.554	34.271	45.880	46.026
**Pandemic hospital**	9.948	9.959	41.427	41.612	42.667	42.573

The means were adjusted for working hours (overtime h/week), age, education level, sex, working experience, marital status, and working place.

## Data Availability

The data presented in this study are available on request from the corresponding author. The data are not publicly available due to privacy issues.
